# Anti-Leukemia Activity of MS-275 Histone Deacetylase Inhibitor Implicates 4-1BBL/4-1BB Immunomodulatory Functions

**DOI:** 10.1371/journal.pone.0007085

**Published:** 2009-09-17

**Authors:** Bérengère Vire, Stéphane de Walque, Audrey Restouin, Daniel Olive, Carine Van Lint, Yves Collette

**Affiliations:** 1 INSERM U891, Centre de Recherche en Cancérologie de Marseille, Marseille, France; 2 Institut Paoli-Calmettes, Marseille, France; 3 Université de la Méditerranée, Marseille, France; 4 Université Libre de Bruxelles, Institut de Biologie et de Médecine Moléculaires (IBMM), Laboratoire de Virologie Moléculaire, Gosselies, Belgique; Johns Hopkins School of Medicine, United States of America

## Abstract

Histone deacetylase inhibitors (HDACi) have demonstrated promising therapeutic potential in clinical trials for hematological malignancies. HDACi, such as SAHA/Vorinostat, Trichostatin A, and MS-275 were found to induce apoptosis of leukemic blasts through activation of the death receptor pathway and transcriptional induction of the Tumor Necrosis Factor (TNF)-related pro-apoptotic family members, TRAIL and FasL. The impact of HDACi on TNF-related costimulatory molecules such as 4-1BB ligand (4-1BBL/*TNFSF9*) is however not known. Following exposure to SAHA/Vorinostat, Trichostatin A, and MS-275, transcript levels were determined by real time PCR in Jurkat, Raji and U937 cells. Treatment with HDACi up-regulated *TNFSF9* gene expression in the three leukemia cell lines, yet to different extend and with distinct kinetics, which did not require *de novo* protein synthesis and was not associated with DNAse I hypersensitive chromatin remodeling. Transcriptional activity of *TNFSF9* promoter-luciferase constructs was induced up to 12 fold by HDACi, and implication of Sp1/Sp3 transcription factors binding to functional GC-box elements was evidenced by reporter gene assays, site-directed mutagenesis, and electrophoretic mobility shift assays. Functionality of modulated target genes was assessed in allogeneic mixed leukocyte reaction experiments. MS-275- and to a lesser extent Trichostatin A- and SAHA-treated Raji cells significantly up regulated T lymphocytes proliferation which was reduced by about 50% by a 4-1BB blocking recombinant protein, while MS-275- but neither Trichostatin A- nor SAHA-treated cells up-regulated IFNγ secretion by T lymphocytes. Our results identify 4-1BBL/4-1BB as a downstream target of HDACi, especially of MS-275 anti-leukemia action *in vitro*. Thus, HDACi such as MS-275 displaying dual TNF-dependent proapoptotic and costimulatory activities might be favored for inclusion in HDACi-based anti-cancer therapeutic strategies.

## Introduction

Members of the Tumor Necrosis Factor ligands and receptors Superfamily (TNFSF) are both secreted and membrane-bound factors that regulate proliferation, activation, differentiation, maturation and survival or programmed cell death of lymphoid, myeloid and other haematopoietic, as well as various non-haematopoietic cell types [1], [2]. TNFSF are also implicated in various acquired or genetic diseases, and have been diversely involved in the control of carcinogenesis. While TNFα or TNFR1,2/TNFRSF1A,1B targeting induce tumor development resistance [3], [4], TRAIL/TNFSF10 deficiency accelerates hematological malignancies [5]. Furthermore, several TNFSF have demonstrated significant antitumor potential in a number of pre-clinical murine and human tumor models [6]. Notably, the finding that agonistic 4-1BB monoclonal antibodies can greatly enhance the expansion of CD8^+^ T cells *in vivo* and eradicate established tumors raised great interest in 4-1BB as a therapeutic target [7], [8]. Hence, strategies that aim to control TNFSF functions are intensively pursued, including the use of recombinant proteins, specific monoclonal antibodies, and by gene transfer. Recently, the tumor-selective action of histone deacetylase inhibitors (HDACi) was shown to involve TRAIL induction in acute myeloid leukemia cells [9], [10], paving the road to drug-based antitumor therapeutic strategies targeting TNFSF functions.

HDACi represent a new class of antitumor agents acting on histone deacetylase (HDAC) enzymatic activity [11], [12]. Despite the widespread role of HDAC in the transcriptional regulation of gene expression, HDACi are relatively non-toxic to normal cells both *in vitro* and *in vivo*, allowing to their use in anti-cancer therapeutic strategies. However, it remains unclear how HDACi exert this cancer cell-selective activity. HDACi are able to induce cell cycle arrest, differentiation, and both intrinsic and extrinsic apoptotic cell death of tumor cells [11]–[13]. Many HDACi have been identified, including the hydroxamate compounds Trichostatin A (TSA) and Suberoylanilide hydroxamic acid (SAHA) that are potent nanomolar HDACi, and the benzamide derivative MS-275, a non hydroxamate micromolar inhibitor of HDACs [13]–[16]. Previous reports have shown that these HDACi up-regulate transcription of the p21/*WAF/CDKN1A* cell-cycle inhibitor and induce *TNFSF10* transcription, accounting for the *in vitro* HDACi-induced G1 arrest and extrinsic apoptosis, respectively [9], [10]. *In vivo*, knocking down *TNFSF10* impairs the antitumor effect of MS-275 [10], but antitumor activity of HDACi may further involve additional biological effects such as reduced angiogenesis [17] and inflammation [18], as well as increased immunogenicity [19], [20].

In this study, we identified *TNFSF9/4-1BBL* as a HDACi target that can mediate anti-leukemia allogeneic leukocyte response.

## Materials and Methods

### Cells, Culture condition

Jurkat T cells (JA16 clone), Raji cells and U937 cells were maintained in RPMI 1640 medium supplemented with 10% fetal calf serum. *Drosophila melanogaster* SL2 cells were maintained in Insect-X-Press medium (BioWhittaker) supplemented with 10% FCS at 25°C without CO_2_.

### HDACIs and other reagents

Three HDACi were used in this study: Trichostatin A (TSA) was obtained from Sigma, MS-275 and SAHA were obtained from Alexis. Cycloheximide (CHX) and Mithramycin A (MA) were obtained from SIGMA.

### Plasmid constructs

The primers used for the p*TNFSF9* constructs were:

p*TNFSF9* (1), 5′-CAGGGAGAGAGACACAGAGACAGAG-3′;

p*TNFSF9* (2), 5′-GATCTCTTTCCACCCACTGCAGAGGC-3′;

p*TNFSF9* (3), 5′-GACGAGGGGAAAGGCTCTGGG-3′;

p*TNFSF9* (4), 5′-GGCCTCCTTTTGTAGCCAAGCAGC-3′

and the 3′ primer: 5′-GACGAGAGACTGCGGGAAGACACAGC-3′. The PCR products were cloned into the reporter vector pGL3-Basic adapted to the Gateway technology (a gift from T.Virolle). Whole nucleotide sequences from these constructs were confirmed by sequencing.

### Site-directed Mutagenesis

p*TNFSF9* (3) was used as a template for mutagenesis performed by the QuickChange site-directed mutagenesis method (Stratagene). The primers used to obtain Sp points mutants are:

m1, 5′-GGAAAGGCTCTG**TT**CTGGGAAG**TT**GCGTG**T**CCGCGGGCGGAGG-3′;

m2, 5′-GGGCGTGGCCGCG**TT**CGGAG**TT**GCGT**TT**CCGCGGGCGGAGG-3′;

m3, 5′-GGCGTGGCCGCG**TT**CGGAG**TT**GCGT**T**GCCTCCTTTTGTAGCC-3′.

Mutations were confirmed by DNA sequencing.

### RNA extraction and RT-PCR

Total RNA was isolated from the cells using the RNeasy mini kit (Qiagen) according to the manufacturer’s protocol. 2 µg of total RNA was reversed transcribed into cDNA. The expression of *TNFSF9* and *GAPDH* mRNA was analyzed by RT-PCR using the following primers for *TNFSF9*: forward, 5′-GTTTCACTTGCGCTGCACCTGCAGCCACTG-3′ and reverse, 5′-TATCAACGTCCAACTTGGGGAAGG-3′; and for *GAPDH*, forward, 5′-GTCATCCCTGAGCTGAAC-3′ and reverse, 5′-GGGTCTTACTCCTTGGAG-3′. Amplification was performed with denaturation at 94°C for 50 s, annealing at 60°C (for *GAPDH*) or 65°C (for *TNFSF9*) for 45 s and extension at 72°C for 45 s. The PCR products for *TNFSF9* (466 bp) and *GAPDH* (613 bp) were separated by electrophoresis on a 3% agarose gel and visualized by staining with ethidium bromide.

### Quantitative RT-PCR

Primer pairs for seventeen TNFSF ligands and twenty-four receptors were incorporated into a low-density array (Assay on Demand, Applied Biosystems). Three endogenous controls were added to the assay set. Three genes were added that represent genes previously described as being either up- or down-regulated by HDACi (see Table 1 for a complete list of genes included in this array). PCR was developed as recommended by the manufacturer. Briefly, 5 µl cDNA (equivalent to 100 ng of total RNA) was mixed with TaqMan Universal Mix (Applied Biosystems) and loaded into 1 sample port. Thermal cycler conditions were as follows: 2 minutes at 50°C, 15 s at 95°C, 60 s at 60°C for 40 cycles. Capture of fluorescence was recorded on the ABI Prism 7900HT scanner, and the C_T_ was calculated for each assay using Sequence Detection System Software 2.1 (Applied Biosystems). Normalization of quantitative-PCR assays was conducted using the C_T_ value of the *GAPDH* endogenous control. Samples were then converted to a fold change ratio described using standard Δ C_T_ formula where ΔC_T_ = C_T_ target – C_T_ average endogenous controls. Thereafter, ΔΔC_T_ values were calculated by subtracting the ΔC_T_ value of each target from the ΔC_T_ of the calibrator (untreated samples). Clustering of quantitative-PCR data was conducted by Pearson correlation and visualized using the program TIGR Multiexperiment Viewer (MeV) (http://www.tigr.org/software/tm4/mev.html) [47].

**Table 1:** List of genes included in the quantitative RT-PCR low-density array.

Ligands	Receptors	*Housekeeping genes*	*Control genes*
LTα	TNFR1	β-Actine	p21WAF1/CIP1
TNFα		GAPDH	IFNγ
LTβ	LTβR	HPRT	ErbB2/Her2
OX40L	OX40		c-Myc
CD40L	CD40		
FasL	Fas		
CD27L	CD27		
CD30L	CD30		
4-1BBL	4-1BB		
TRAIL	DR4		
	DR5		
	DcR1		
	DcR2		
RANKL	RANK		
	OPG		
TWEAK	Fn14		
APRIL	TACI		
BAFF	BAFFR		
LIGHT	HVEM		
TL1A	DR3		
GITRL	GITR		
	TROY		
	EDAR		

Real time quantitative PCR was performed using a LightCycler rapid thermal cycler (Roche) according to the manufacturer's conditions. Primers for *TNFSF9* and *GAPDH* used were from Applied Biosystems (TaqMan® Gene Expression Assays).

### Flow cytometry analysis

The cells were treated with HDACi for 4, 8, 24 and 48 h before flow cytometric analysis. Briefly, 2×10^5^ cells were incubated with anti-mouse IgG-PE (Beckman Coulter) or anti-4-1BBL antibodies (BD Pharmingen) at 4°C for 30 minutes. After three washes with phosphate-buffered saline (PBS) supplemented with 2% FCS, the cells were subjected to flow cytometric analysis. Samples were analyzed on a FACScan (Becton Dickinson) and analyzed by CellQuest software.

### Nuclease digestion of purified nuclei and southern blotting

Nuclei were purified and submitted to nuclease digestion as previously described [21]. Briefly, cells were lysed at 4°C in 0.2% NP40 buffer followed by nuclease digestion using DNase I (10 min at 4°C). Proteinase K-treated DNA was next purified by phenol extraction. Purified DNA (30 µg) was digested with Sac I and the fragments generated were separated by electrophoresis. Each size marker was generated by digesting genomic DNA (10 µg) with restriction enzymes as indicated in figure legends. After transfer to nylon membranes (Hybond-N+, Amersham Pharmacia Biotech), DNA was UV-cross-linked and membranes were prehybridized, as previously described [21]. The specific probes were synthesized by PCR using the following primer pairs: 5′-CTGCTGATCGATGGGCCCCTGAGC-3′ and 5′-GACCTCGGTGAAGGGAGTCCGGCTG-3′. After random primer labeling, denatured DNA probes were allowed to hybridize for at least 16 h at 68°C followed by extensive washes and autoradiography.

### Electroporation and Luciferase Assays

10^7^ Jurkat T cells were electroporated using Bio-Rad gene pulser II (250 V, 25 ms) with 10 µg of pGL3 or the various *TNFSF9* promoter-driven firefly luciferase constructs together with 5 µg of beta-globine Renilla luciferase plasmid (pRL-β). The cells were incubated for 1 h before they were collected, washed in PBS and lysed to determine the luciferase activity by using the dual luciferase reporter assay according to manufacturer's instructions (Promega), and read using a luminometer (Dynex). The transfection efficiency was normalized to Renilla luciferase activity and corrected for protein content as determined by the Bradford protein assay (Bio-Rad). The reported values represent the average of three independent transfections, with standard deviation as errors bars.

SL2 cells (2×10^5^) were plated in 24-well plates and transfection was carried out using Effectene Transfection Reagent (Qiagen). Each well was transfected with 200 ng of reporter plasmid and with pHMW-Sp1 (0 to 600 ng) supplemented, when necessary, with empty vector (pHMW) to equalize the total DNA transfected to 1 µg. Fourty-eight hours after transfection, cells were harvested and assayed for luciferase activity as described above. Human pHMW-Sp1 and its control vector (pHMW) were gifts from Dr Murphy, T.D, Carnegie Institution of Washington, USA.

### Allogeneic mixed leukocyte reaction

Raji cells were treated for 24 hours with TSA (250 nM), MS-275 (2.5 µM) or SAHA (2.5 µM). After irradiation, 1×10^5^ stimulator B lymphomas cells (Raji) were added to 2×10^5^ PBMCs in each well of 96-well U-bottomed culture plates. OKT3 (0.5 µg/ml) was immobilized onto the surface of the plates (ON, 4°C). Cells were cultured in presence or absence of 1 µg/ml recombinant human 4-1BB-Fc and DR3-Fc (R&D systems) or control Nectine 4-Fc (a gift from M.Lopez). Recombinant proteins were added to the cultures at the same time as cells. Lymphocyte proliferation after 5 days of culture was measured by adding [^3^H]-thymidine (0,2 µCi/well) to the wells for the last 18 h and the cells were harvested. [^3^H] incorporation was determined using a TopCount scintillation counter (Packard Instrument).

### Cytokine assay

IFN-γ levels were measured by ELISA using a cytokine detection kit (BD Biosciences) according to the manufacturer's instructions.

### Electrophoretic mobility shift assays

Nuclear extracts were prepared by a rapid method described by Osborn et al [22]. All buffers contained complete protease inhibitors (Roche). Protein concentrations were determined by the method of Bradford [23]. The DNA sequences of the coding strand of the double-stranded oligonucleotides used for this study are listed in Table 2. Electrophoretic mobility shift assays (EMSAs) were performed as described previously [24]. Briefly, nuclear extract (10 µg of protein) was first incubated on ice for 10 min in the absence of probe and specific competitor DNA in a 16 µl reaction mixture containing 10 µg of DNase-free BSA (Amersham Biosciences), 1–2 µg of poly(dI–dC) (Amersham Biosciences) as non-specific competitor DNA, 50 µM ZnCl2, 0.25 mM DTT, 20 mM HEPES (pH 7.3), 60 mM KCl, 1 mM MgCl2, 0.1 mM EDTA and 10% (v/v) glycerol. 20 000 c.p.m. of probe (10–40 fmol) was then added to the mixture with or without a molar excess of an unlabeled specific DNA competitor, and the mixture was incubated for 20 min on ice. Samples were subjected to electrophoresis at room temperature on 6% polyacrylamide gels at 120 V for 2–3 h in 1x TGE buffer (25 mM Tris–acetate, pH 8.3), 190 mM glycine and 1 mM EDTA). Gels were dried and autoradiographed for 24–48 h at −70°C. For supershift assays, polyclonal antibodies against Sp1 (sc-059X), Sp2 (sc-643X), Sp3 (sc-644X), Sp4 (sc-645X), (Santa Cruz Biotechnology), or a purified rabbit immunoglobulin (IgG) were added to the reaction mixture and incubated for 30 min on ice before the addition of the radiolabeled probe.

**Table 2:** Primers used for Electrophoretic Mobility Shift Assays.

GC box1 wt: 5′-GGA AAG GCT CTG GGC TGG GAA GGG GCG TGG CCG CGG-3′
GC box1 mut: 5′-GGA AAG GCT CTG TTC TGG GAA GTT GCG TGT CCG CGT-3′
GC box2 wt: 5′-TGG CCG CGG GCG GAG GGG CGT GGC CGC GG-3′
GC box2 mut: 5′-TGT CCG CGT TCG GAG TTG CGT TTC CGC GT-3′
GC box3 wt: 5′-TGG CCG CGG GCG GAG GGG CGT GGC CTC CTT-3′
GC box3 mut: 5′-TTT CCG CGT TCG GAG TTG CGT TGC CTC CTT-3′

(Only forward primers are indicated).

## Results

### Up-regulation of TNFSF9 mRNA in HDACi-stimulated leukemia cell lines

**Figure 1:**
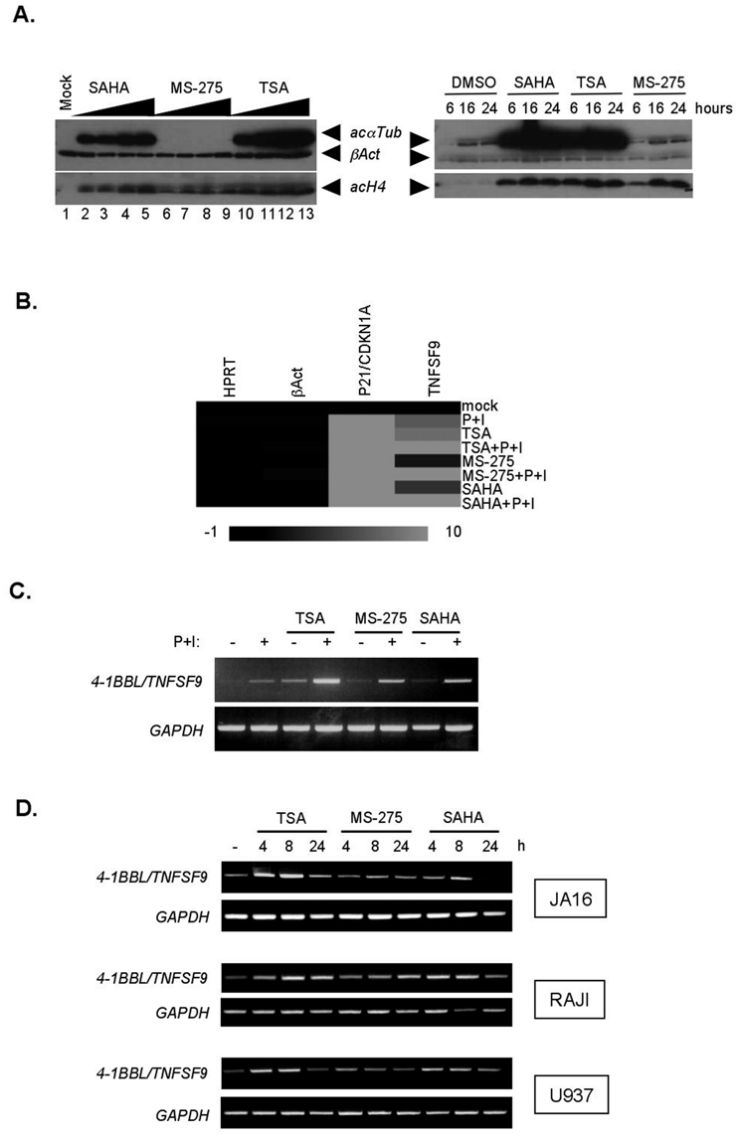
Analysis of *TNFSF9* gene expression profiles induced by HDACi in leukaemia cell lines. (A) Jurkat JA16 cells were incubated with SAHA (lanes 2–5; 0.5, 1, 2.5 and 5 µM), MS-275 (lanes 6–9; 0.5, 1, 2.5 and 5 µM) or TSA (lanes 10–13, 50, 100, 200, 500 nM), or mock-treated (lane 1) for 16 hours, followed by cell lysis, SDS-PAGE and immunoblotting using the indicated antibodies (left panel). In parallel, Jurkat JA16 cells were incubated with SAHA (2 µM), TSA (200 nM), MS-275 (2 µM) or mock-treated (DMSO) for 6, 16 or 24 hours, as indicated, followed by cell lysis, SDS-PAGE and immunoblotting using the indicated antibodies (right panel). (B) Jurkat JA16 cells were incubated with TSA (500 nM), MS-275 (2,5 µM) or SAHA (2,5 µM) for 8 hours and left unstimulated or stimulated by PMA (20 ng/ml) and Ionomycin (1 µg/ml) for 4 hours. Following incubation time, total mRNA was extracted and analyzed by Transcription Low Density Array. Results obtained for *HPRT*, *βActin*, *p21/WAF/CDKN1A* and *TNFSF9* are presented as expression ratios relative to *GAPDH* transcript levels. The scale shows the level of expression, where red indicates increased gene expresssion, and the intensity of color correlated to the magnitude change. Black indicates no change. (C) mRNA from (B) were analyzed by RT-PCR, using *TNFSF9* and *GAPDH* primer pairs. (D) Jurkat JA16, RAJI and U937 cells were mock-treated or incubated with TSA (250 nM), MS-275 (1 µM) and SAHA (1 µM) for 4, 8 or 24 hours. Total mRNA was extracted and transcripts of *TNFSF9* and *GAPDH* were analyzed by RT-PCR. These conditions were compromized to compare for *TNFSF9* transcript detection and modulation by HDAC inhibition in different cell types producing distinct basal *TNFSF9* transcript levels.

Modification of histone and α tubulin acetylation by HDAC inhibition was first investigated in the Jurkat T cell leukemia cell lines using different treatment times and dosages of the hydroxamic-derived Trichostatin A (TSA), the Suberoylanilide hydroxamic acid (SAHA), and the benzamide-derived MS-275 HDACi. As shown in Fig. 1A, while the three HDACi induced dose-dependent acetylation of histone H4, which peaked at 16 hours of treatment, only SAHA and TSA induced α tubulin acetylation, as expected from the known incapacity of MS-275 to impact on the specific acetylation of α tubulin by HDAC 6.

These experimental conditions were developped to investigate for the transcriptional modulation of genes of the TNFSF familly using a transcription low-density array (see list of genes in Table 1). Modification of *TNFSF* mRNA profiles induced by 8 h treatment with TSA, SAHA, and MS-275 were determined in Jurkat cells, followed or not by a 4 h stimulation by a combination of phorbol ester (PMA) and calcium ionophore (Ionomycin) to up-regulate gene transcription. As compared to untreated cells, each HDACi strongly induced p21/*WAF/CDKN1A* cell cycle inhibitor, used here as control for HDACi-regulated gene transcription (Fig. 1B). Significant (above 2-fold and up to 15-fold) modifications of *TNFSF* transcript levels were also noted, consistent with previous reports (not shown [12], [25]). Interestingly, *TNFSF9* was the only *TNFSF* out of the 17 *TNFSF* present in the array to be modulated by the three HDACi (not shown). Indeed, as showed in Fig. 1B, the three investigated HDACi up-regulated mRNA levels of *TNFSF9* by two- to eight-fold, but not *HPRT* and *β Actin* mRNA levels, as normalized to *GAPDH* levels. Transcriptional induction by the combination of PMA and Ionomycin further increased HDACi-induced *TNFSF9* mRNA levels, which was confirmed by RT-PCR analysis (Fig. 1C), as well as by quantitative real time RT-PCR (data not shown). Time course RT-PCR experiments further showed that *TNFSF9* mRNA levels were up-regulated by the three HDACi in Jurkat and also in the Raji Burkit B lymphoma and U937 myeloid cell lines, in a time-dependent manner, as compared to endogenous *GAPDH* levels, yet with distinct kinetics (Fig. 1D).

### Up-regulation of TNFSF9 by HDACi does not require de novo protein synthesis and is not associated with DNAse I hypersensitive chromatin remodeling

**Figure 2:**
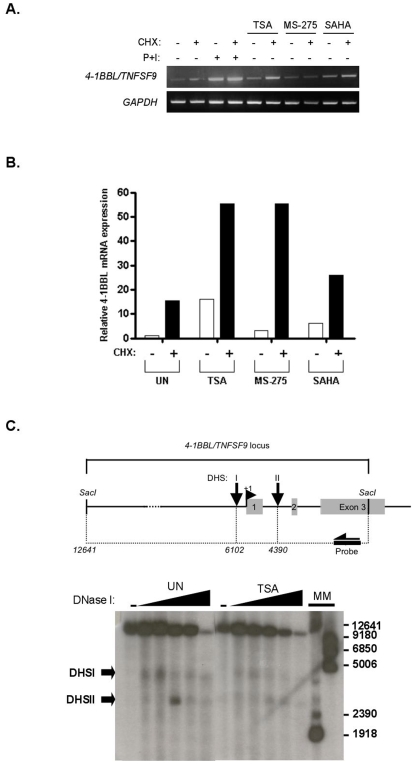
Modulation of *TNFSF9* transcript levels by HDACi does not require *de novo* protein synthesis and does not require *TNFSF9* promoter region chromatin remodeling. Jurkat JA16 cells were left unstimulated (UN) or were incubated for 1 hour with CHX (10 µg/ml) and then treated for 8 hours by TSA (250 nM), MS-275 (1 µM) and SAHA (1 µM). Total mRNA was extracted and transcripts of *TNFSF9* and *GAPDH* were analysed by PCR (A) and quantified by real-time PCR (B) as described in Fig. 1. (C) Nuclei from Jurkat T cells left unstimulated (UN), or treated with TSA (500 nM) for 18 hours, were digested *in vivo* with increasing amounts of DNase I (0, 30, 40, 50, 60 and 70 U/ml) followed by *Sac*I digestion *in vitro* and indirect end-labeling (lower panel). The schematic organization of the *TNFSF9* locus, showing the indirect end-labeling and southern blotting strategy, as well as the DNaseI hypersensitive sites (DHS) identified in the course of the present study are depicted in the upper panel. The position of the probe used for the southern blotting, as well as the *TNFSF9* exon 1-3 are showed. Hypersensitive regions DHSI and DHSII are shown on the left. Molecular weight markers (MM) are a double digest of naked DNA by *Sac*I (12641 bp), *Nsi*I (9180 bp), *Nhe*I (6850 bp), *Hinc*II (5006 bp), *Xho*I (2390 bp) and *Bgl*II (1918 bp).

To explore the mechanism of HDACi-induced *TNFSF9* up-regulation, Jurkat cells were pre-incubated with the protein synthesis inhibitor, cycloheximide (CHX) followed by further incubation for 8 h in the presence or absence of HDACi. As shown in Fig. 2A, *TNFSF9* mRNA levels were not reduced by CHX treatment, but instead were observed to be markedly up-regulated. This was further confirmed by QRT-PCR (Fig. 2B), suggesting that *TNFSF9* mRNA levels are regulated by neosynthesized cellular factors. However, this effect appeared to not be modulated by HDAC inhibition (Fig. 2A and 2B), indicating that the observed upregulation of *TNFSF9* mRNA levels by HDACi does not appear to require neosynthesized cellular factors, and suggests the direct implication of transcriptional regulatory mechanisms. In eukaryotes, chromatin is recognized as an important modulator of transcriptional regulatory mechanisms [26]. Increased histone acetylation, such as following HDAC activity pharmacological inhibition, neutralizes the positive charge of lysine residues, allowing a more “open” chromatin structure that facilitates transcription. We thus investigated whether the upregulation of *TNFSF9 mRNA levels by* HDACiinvolved chromatin remodeling. DNAse I digestion of the *TNFSF9* locus followed by indirect end-labeling identified two major dose-dependent DNase I hypersensitive sites (DHSI and II) that were mapped to the 5′ regulatory promoter region and between exon 1 and exon 2 (Fig. 2C), respectively. This pattern remained unchanged following HDACi treatment, indicating that the modulation of *TNFSF9* mRNA levels by HDACi expression does not involve detectable DNAse I hypersensitive chromatin remodeling within the region under study, however, this study does not exclude localized promoter architecture modifications. The hypersensitivity of the promoter region to DNAse I digestion in unstimulated cells correlated with the basal constitutive transcription of *TNFSF9* in Jurkat T cells (data not shown).

### Identification and mapping of HDACi responsive elements within the TNFSF9 promoter region

**Figure 3:**
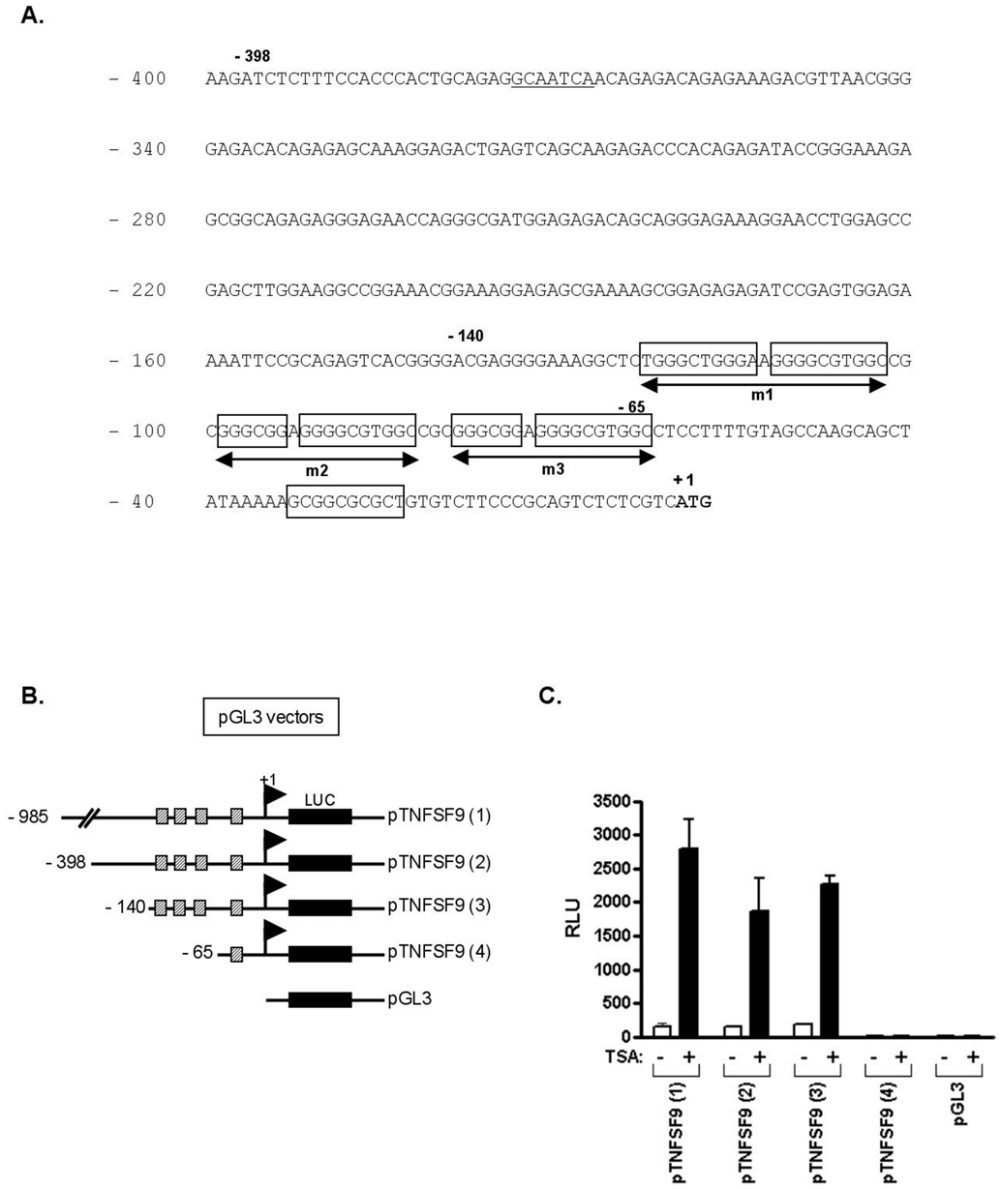
Identification of TSA response elements in the human *TNFSF9* promoter. (A) DNA sequence of a 400-bp human *TNFSF9* promoter region. The ATG site is *boldface* (to which we assigned the position nucleotide + 1). Putative Sp1/Sp3 binding sites are boxed. Putative CCAAT boxes are underlined. (B) The constructs were generated by cloning progressively 5′-truncated human *TNFSF9* promoter fragments into the pGL3/basic luciferase vector. Negative numbers denote bp distances from translational start codon. (C) Jurkat cells were transiently cotransfected with the indicated reporter constructs and pRL-β to control for transfection efficiency. Transfected cells were either left untreated or treated with TSA (500 nM) for 16 hours. Data are the average ± SD of three independent experiments. *RLU*, relative light units.

To determine whether HDACi activate *TNFSF9* transcription, we isolated human genomic fragments containing the predicted *TNFSF9* 5′ regulatory regions (Fig. 3A). These genomic DNA fragments were inserted upstream to the luciferase reporter gene in the pGL3 basic plasmid construct (Fig. 3B). Promoter activity of the corresponding constructs (p*TNFSF9* (1)-(4)) was assayed by measuring firefly luciferase activity after transient transfection in Jurkat T cells (Fig. 3C). p*TNFSF9*(1), p*TNFSF9*(2) and p*TNFSF9*(3) transcriptional activity was induced up to 12 fold by TSA treatment, indicating the presence of TSA-inducible element(s) within the 985, 398 and 140 bp genomic DNA fragment controlling firefly luciferase transcription (Fig. 3C). In contrast, firefly luciferase activity was not induced by TSA treatment of cells transfected with the p*TNFSF9*(4) or pGL3 basic empty constructs, indicating that these TSA-inducible element(s) are contained between nucleotide –140 and –65 according to the translation initiation site (Fig. 3). Similar results were observed using different HDACi in different cell lines (data not shown).

### Identification of HDACi responsive elements within the TNFSF9 promoter region

**Figure 4:**
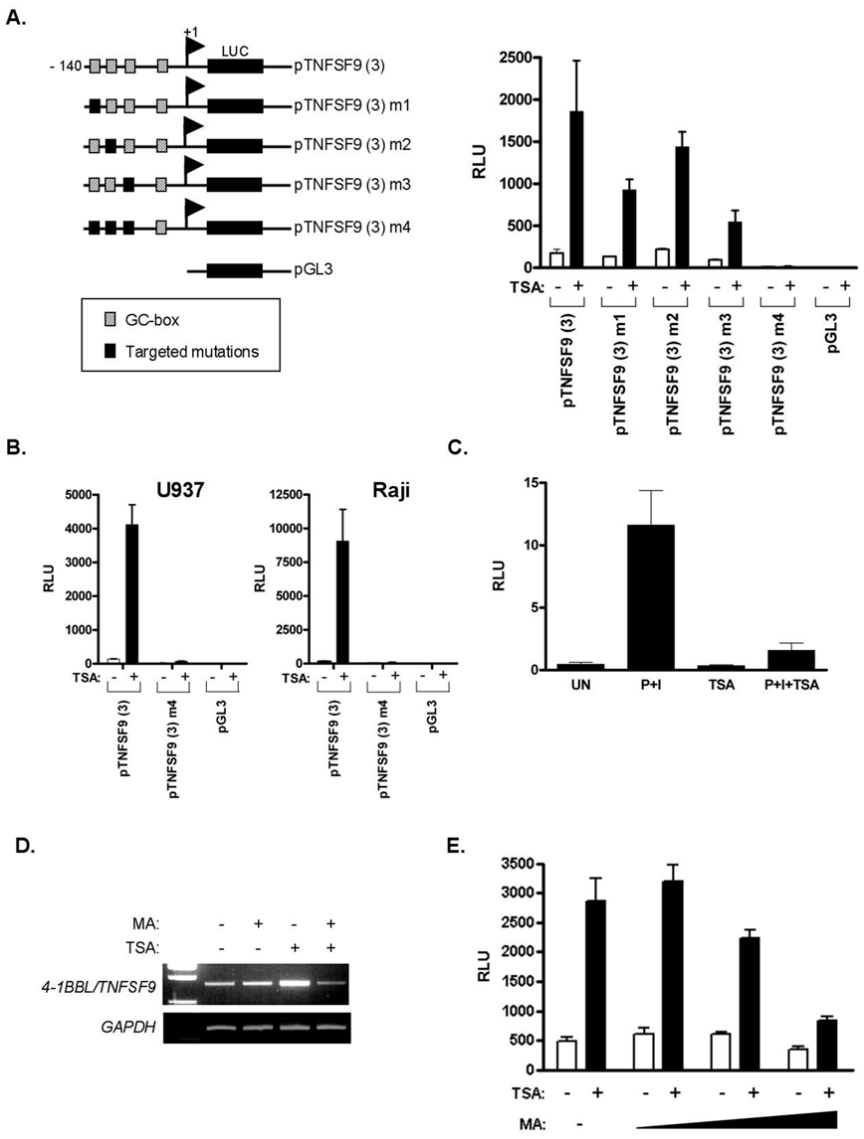
TSA-induced *TNFSF9* promoter activity depends on GC-boxes sequences. (A) The different pTNFSF9 (3)-luc mutated constructs were generated as described under “Materials and Methods”. The *hatched* boxes represent putative GC-boxes. The mutations in theses sites are symbolized by *filled* boxes. Jurkat cells were transiently cotransfected with the indicated reporter construct and the pRL-β to control for transfection efficiency. Cells were left unstimulated or treated with TSA (500 nM) for 16 hours. Means ± SD of three independent experiments are shown. Basal transcriptional activities were 7.7±1.8, 13.5±4.9, 96.7±19.2, 222.2±20.3, 133.5±7.6 and 175±81.7 RLU for the pGL3, pTNFSF9 (3) m4, pTNFSF9 (3) m3, pTNFSF9 (3) m2, pTNFSF9 (3) m1 and pTNFSF9 (3), respectively. (B) The indicated pTNFSF9 (3)-luc constructs were transfected in U937 (left panel) and Raji (right panel) cell lines and analyzed as described in (A). (C) The p-486TNFSF6/FasL promoter construct was transfected in Jurkat cells and analyzed as described in (A) except that PMA (20 ng/ml) and Ionomycin (1 µg/ml) were added, as indicated. Means ± SD of three independent experiments are shown. (D) RT-PCR was performed on total mRNA isolated from Jurkat cells pretreated with the GC-box binding inhibitor mithramycin A (MA) for 1 hour followed by incubation in the presence TSA (250 nM) for 8 hours. (E) Increasing concentrations of MA (100, 200 and 500 nM) was added to the cells 1 hour after transfection with the p*TNFSF9* (3) reporter plasmid, followed by the addition of TSA (500 nM) 1 hour later. The cells were harvested 16 hours after the addition of TSA and processed for luciferase assay. Means ± SD of three independent experiments are shown.

Previous studies have shown that conserved cis-regulatory elements, particularly Sp1 and CCAAT boxes, located in the promoters of several genes are responsible for transcriptional activation by HDACi [27]–[32]. Sequence analysis of the *pTNFSF9* region contained between the nucleotides −140 and −65 upstream to the translation initiation site using MatInspector (Genomatix Software, Munich, Germany) and TESS (Transcription Element Search System, CBIL, US) allowed the identification of consensus binding sites for transcriptions factors of the Sp (GC-box) family (Fig. 3A). Mutations of the individual GC-box, as well as combined mutations were performed to evaluate the functional significance of these putative Sp binding sites. As shown in Fig. 4A, individual mutations did not significantly alter basal promoter activity, yet TSA-activated p*TNFSF9*(3) promoter luciferase activity was reduced by about 25–70% in Jurkat T cells, depending on the individual mutated GC-rich sequence, whereas the combined mutation (p*TNFSF9*(3) m4) abolished both the basal- and the TSA-induced activity, as compared to the control pGL3 basic reporter construct. Similar results were obtained upon transfection of the p*TNFSF9*(3) and p*TNFSF9*(3) m4 promoter constructs in the U937 and Raji cell lines (Fig. 4B). These results identify the GC-boxes studied as important mediators of HDACi inducibility of *TNFSF9* promoter activity. To gain more insights into the specificity of GC-rich sequences in TSA-induced transcription, a previously described p-486TNFSF6/FasL promoter construct containing functional GC-rich sequences was transfected into Jurkat cells and analyzed for its responsiveness to TSA treatment ([21], [33]). As shown in Fig. 4C, despite transcriptional induction of this promoter construct by PMA plus Ionomycin treatment, TSA did not stimulate p-486TNFSF6/FasL-mediated transcription. On the opposite, TSA co-treatment down-regulated PMA plus Ionomycin-induced transcription, indicating that the presence of functional GC-rich sequences does not predict TSA-responsiveness (Fig. 4B).

### Effect of the mithramycin A DNA intercalating agent on HDACi-induced TNFSF9 gene expression

Mithramycin A is an anti-tumor antibiotic which inhibits transcription from promoters containing GC-rich DNA sequences [34], [35]. We thus evaluated HDACi-induced *TNFSF9* expression following mithramycin A treatment. As shown in Fig. 4C, mithramycin A prevented TSA-induced *TNFSF9* mRNA up-regulation. Consistent with the decreased levels of *TNFSF9* mRNA, mithramycin A also reduced TSA-induced p*TNFSF9*(3)-mediated transcriptional activity in a dose-dependent manner (Fig. 4D). Collectively, this data strongly indicates that GC-box interacting transcription factors are involved in HDACi-induced *TNFSF9* expression.

### Sp1/Sp3 transcription factors bind to TNFSF9 promoter GC box elements in vitro and increase its promoter activity

**Figure 5:**
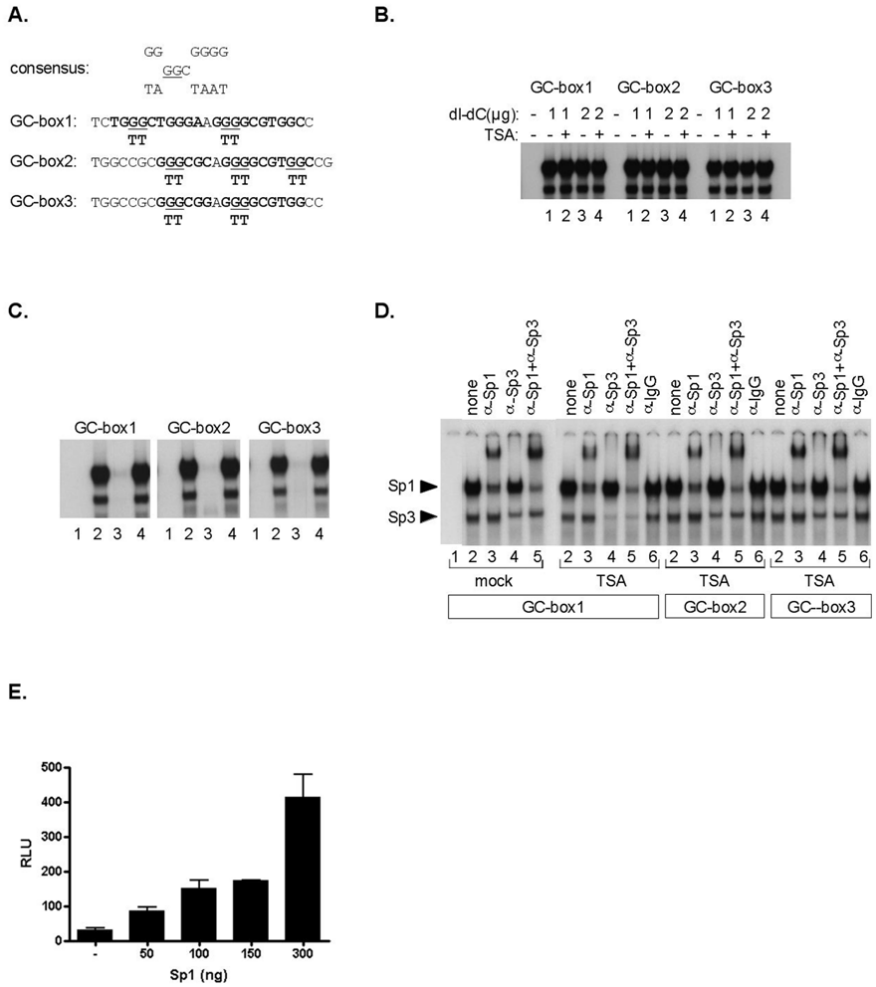
Sp proteins bind to the GC-box located in the *TNFSF9* promoter. (A) Nucleotide sequence of the three wild-type GC-box oligonucleotides probes are shown with underlined bases corresponding to the mutated bases. (B) The three wild-type GC-box oligonucleotides probes were incubated with nuclear extracts from Jurkat cells (10 µg) and treated or not for 4 hours with TSA (250 nM) in the absence or in the presence of unspecific competitor (dI-dC). The figure shows only the specific retarded bands of interest. (C) The three wild-type GC-box oligonucleotide probes were incubated with nuclear extracts from Jurkat cells (10 µg) in the absence (lane 2) or in the presence of the unlabeled oligonucleotide (lane 3) or of the mutated unlabeled oligonucleotide (lane 4). The figure shows only the specific retarded bands of interest. (D) Nuclear extracts from Jurkat cells treated or not for 4 hours with TSA (250 nM) were incubated in the absence of antibody or in the presence of antibodies directed against Sp1 and/or Sp3 (as indicated at the top of each lane) or with purified rabbit IgG as negative control, before addition of the oligonucleotide probe. The figure shows only the specific retarded bands of interest. Arrows indicate specific retarded DNA-protein complexes corresponding to Sp1 and Sp3. (E) SL2 cells were transiently cotransfected with pGL3 or p*TNFSF9* (3)-luc and with various amounts of the Sp1 expression construct (pHMW-Sp1) or the empty vector (pHMW) and processed for luciferase assays. Values were normalized relative to the protein concentration of the cellular lyzates. The experiment was repeated three times with similar results. *RLU*, relative light units.

In order to assess the binding of members of the Sp family to the GC-boxes that we identified in the *TNFSF9* promoter region, we designed double-stranded oligonucleotides (Fig. 5A). These oligonucleotides were radiolabeled and tested in electrophoretic mobility shift assays (EMSA) for DNA–protein interactions with nuclear extracts from mock-treated and TSA-treated Jurkat cells (Fig. 5B). Retarded protein–DNA complexes were observed using the three distinct GC-box oligonucleotides and TSA treatment did not induce detectable modification of the observed pattern of retarded protein-DNA complexes. To evaluate the sequence specificity of the binding to these GC-boxes, we performed competition EMSAs using different unlabeled double-stranded competitor oligonucleotides (Fig. 5C). The specificity of the protein binding was demonstrated because their formation was inhibited by competition with molar excesses of the unlabeled homologous oligonucleotides (Fig. 5C, lane 3). In contrast, these complexes were not competed by mutated version of the oligonucleotides (lane 4). Similar results were observed for the three GC-boxes. To identify directly the Sp family members within the retarded complexes, we performed supershift assays using specific antibodies directed against individual members of the Sp family of transcription factors (Fig. 5D). The wild-type probe was incubated with nuclear extracts from Jurkat cells and polyclonal antibodies directed against Sp1 and/or Sp3 were added to the binding reaction mixture. The Sp1 antibody selectively supershifted the major slower migrating complex (Fig. 5D, lane 3) and the Sp3 antibody resulted in the strong decrease of the faster migrating complex (Fig. 5D, lane 4). We confirmed these results when both the anti-Sp1 and anti-Sp3 antibodies were included in the same binding reaction (Fig. 5D, lane 5). In contrast, the binding pattern was not affected by the addition of the antibodies directed against other Sp proteins (Sp2 and Sp4) (data not shown), showing that the two complexes did not seem to involve these other proteins. Moreover, the binding pattern was not affected by the addition of purified IgG, used as a negative control (Fig. 5D, lane 6). Again, similar results were obtained using the three GC-boxes. Of note, TSA treatment did not appear to detectably alter Sp1 and/or Sp3 binding (Fig. 5D). Overall, these results demonstrate that Sp1 and Sp3 transcription factors interact with the GC-box (renamed Sp site hereafter in the manuscript) located in the *TNFSF9* promoter region. SL2 cells are devoid of endogenous human Sp family transcription factors and thus represent a useful model to investigate Sp-dependent transcriptional mechanisms [36], [37]. SL2 cells were cotransfected with the p*TNFSF9*(3) promoter construct along with pHMW-Sp1 (insect expression vectors encoding for human Sp1). Over expression of Sp1 in SL2 cells resulted in an increased expression of the p*TNFSF9*(3) promoter construct in a dose-dependent manner (Figure 5E). Similar results were obtained upon expression of Sp3 transcription factor (not shown).

### HDACi-induced TNFSF9 expression increases anti-leukemia allogeneic leukocyte response

**Figure 6:**
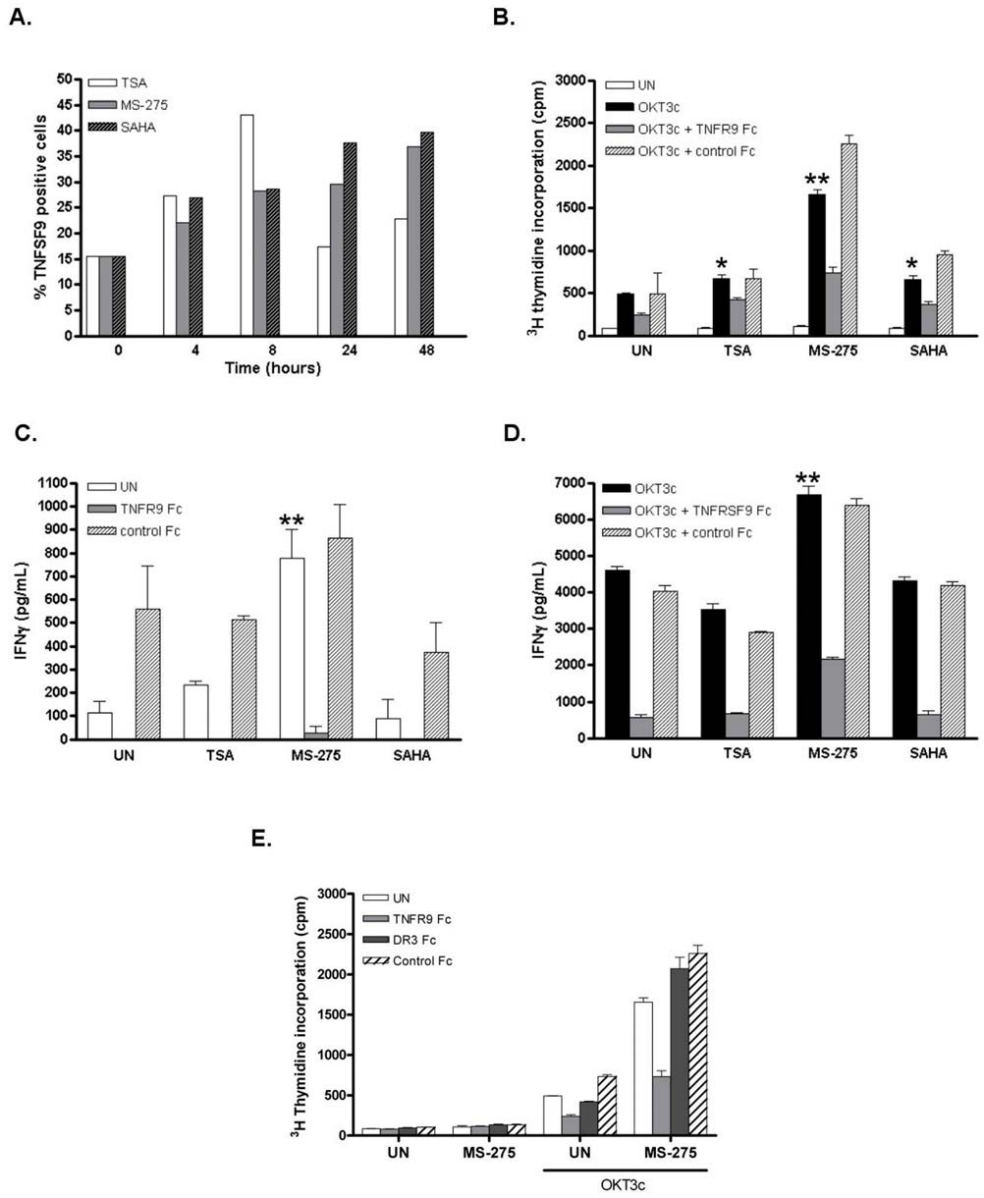
Induced proliferation of normal allogeneic leukocytes by Raji cells treated with HDACi. (A) FACS analysis of 4-1BBL expression on HDACi-treated Raji cells for 4, 8, 24 and 48 hours as indicated. Results are presented as % of positive cells. The data shown are from one experiment representative of a total of two. (B) PBMCs (2×10^5^) of healthy donor were incubated for 5 days with Raji cells (1×10^5^) treated or not for 24 hours with TSA (250 nM), MS-275 (2.5 µM) or SAHA (2.5 µM). PBMCs were stimulated or not with immobilized OKT3 (0,5 µg/ml) in the presence or absence of recombinant human 4-1BB-Fc (1 µg/ml) or control-Fc protein (1 µg/ml), as indicated, and proliferation was measured by incorporation of ^3^H-thymidine during the last 18 hours of culture. Supernatants from unstimulated (C) and from OKT3-stimulated (D) cell cultures were collected at 48 hours of culture and assayed for IFNγ by ELISA. Values are means of triplicates ± SD and this experiment is representative of two independent experiments. * p<0,05 and ** p<0,01 (t student). (E) As in (B), except that DR3-Fc recombinant protein was added (1 µM/ml), as indicated. Values are means of triplicates ± SD.

In an attempt to determine the functional significance of HDACi-induced *TNFSF9* mRNA levels, 4-1BBL protein expresssion levels and function were evaluated. Time course Flow Cytometry experiments evidenced the up-regulation of 4-1BBL cell surface expression by the three HDACi in Raji cells (Fig. 6A). Ralji cells were next incubated in the presence of HDACi for 24 hours followed by γ-irradiation to prevent further cell proliferation and co-culture with purified T lymphocytes from healthy donors. IFNγ secretion was determined by ELISA and T lymphocyte proliferation was assessed by [^3^H]-thymidine uptake. MS-275-stimulated Raji cells significantly up-regulated IFNγ secretion by 7 fold (t student, p<0,01), which was prevented by the recombinant 4-1BB-Fc but not control-Fc protein (Fig. 6B), indicating a requirement for 4-1BB/4-1BBL interaction for IFNγ secretion stimulated by HDACi-treated Raji cells. TcR/CD3 triggering to activate T lymphocytes to up-regulate cell surface 4-1BB [38]–[40], induced a marked up-regulation of IFNγ secretion which was further significantly increased by co-culture of stimulated T cells with MS-275-stimulated Raji cells as compared to untreated Raji cells (6675,8±409,3 pg/ml *versus* 4607,4±167,4 pg/ml; t student: p<0,01), in a 4-1BB/4-1BBL interaction dependent-manner (Fig. 6B). In contrast to MS-275, TSA- and SAHA-stimulated Raji cells did not stimulate increased IFNγ secretion (Fig. 6B). However, HDACi-stimulated Raji cells significantly up-regulated T cell proliferation, as compared to untreated Raji cells, using either TSA and SAHA (t student, p<0,05) or MS-275 (t student, p<0,01) (Fig. 6C). Addition of the recombinant 4-1BB-Fc but not control-Fc protein efficiently prevented up-regulation of T lymphocyte proliferation by HDACi-stimulated Raji cells, implicating 4-1BB/4-1BBL interactions in the increased proliferation (Fig. 6C). To further examine for the specificity of 4-1BB/4-1BBL interactions in this HDACi-stimulated mixed lymphoid reaction, DR3-Fc recombinant protein was used as an additional control. Indeed, TL1A/TNFSF15-DR3 interactions have been implicated in T cell proliferation and IFNγ secretion [41]. Using the DR3-Fc recombinant protein to block these interactions, only 4-1BB-Fc, but neither the control-Fc nor the DR3-Fc recombinant proteins efficiently prevented up-regulation of T lymphocyte proliferation by HDACi-stimulated Raji cells, specifically implicating 4-1BB/4-1BBL interactions in the increased proliferation (Fig. 6E)

## Discussion

In this study, we identified 4-1BBL/*TNFSF9* as a transcriptionaly activated target of three distinct HDACi in leukemia cell lines and most importantly that MS-275-induced 4-1BB/4-1BBL interactions potentiated anti-leukemia allogeneic leukocyte response, hence providing novel molecular insights into the antileukemia mechanisms of action of HDACi.

Regulation of gene expression is important for HDACi antitumor activities [42]–[44]. Expression profiling studies have identified that limited sets of target genes (2–10%) are regulated by HDACi [45]–[48], yet more recent studies have suggested that this number might have been underestimated [43]. Nonetheless, the molecular basis to explain how HDACi impact gene expression remains poorly understood at the genome level. As previously described for *CDKN1A/p21/WAF* [49], *TNFSF10* [10] and other HDACi-activated genes [50], we show here that the *TNFSF9* promoter region contains functional Sp1/Sp3 transcription factors binding sites that are critical for its HDACi-induced gene transcription. But how Sp1/Sp3 transcription factors modulate *TNFSF9* transcription in an HDACi-regulated manner remains to be established. Using the indirect-end labeling technique, DNAse I hypersensitive sites (DHSI and II) were identified, DHSI being mapped to the *TNFSF9* minimal promoter region containing the Sp1/Sp3 DNA binding sites. DHSI was detected in unstimulated Jurkat cells displaying basal *TNFSF9* transcription and was not detectably altered upon HDACi treatment, in agreement with the transcriptional activation of *TNFSF9* promoter constructs by HDACi and suggesting that HDACi-induced *TNFSF9* transcription did not involve DNase I-sensitive large chromatin remodeling events. The transcriptional activity of Sp1 family members can be modulated by acetylation [13], [51], [52] and both Sp1 and Sp3 can interact with HDAC [53], suggesting a role for Sp1 and/or Sp3 acetylation in the Sp1/Sp3-dependent, HDACi-regulated *TNFSF9* induction. Alternatively, Sp1/Sp3 may recruit acetylated co-factors allowing *TNFSF9* transcriptional induction. To asses the *in vivo* binding of Sp factors to the *TNFSF9* promoter region, we attempted to perform chromatin immunoprecipitation assays. Preliminary experiments showed low enrichement of the *TNFSF9* promoter region in Sp1 immunoprecipitates, as compared to control antibodies, that were also not detectably modified by TSA treatment in agreement with previous observations ((Nebbioso, 2005 #173] and data not shown).

Of interest, nonetheless, mithramycin A abrogated both HDACi-stimulated endogenous and promoter-driven *TNFSF9* transcription, yet it did not affect basal *TNFSF9* transcription, supporting the hypothesis that acetylation of Sp1/Sp3 or associated factors, and not *per se* Sp1/Sp3 chromatin-recruitment, was required for HDACi-regulated *TNFSF9* induction. Consistent with this hypothesis, EMSA experiments did not reveal increased Sp1/Sp3 DNA binding upon HDACi treatment. Collectively, these results support a model where protein acetylation induced by HDACi increases the transcriptional activity of Sp1/Sp3-dependent complexes seated on the *TNFSF9* promoter. The mechanisms of acetylation of Sp1/Sp3 or associated factors in HDACi-induced *TNFSF9* transcription remains to established.

One important finding of the present study was the functional up-regulation of 4-1BBL by MS-275, as evidenced by enhanced T cell proliferation and IFNγ production in mixed peripheral blood leukocytes co-culture experiments. While TRAIL up-regulation was convincingly shown to contribute to the selective HDACi-induced apoptosis of tumor cells, our results provide first evidence that up-regulated 4-1BBL might further contribute to the *in vivo* anticancer action of HDACi such as MS-275. There has been ample evidence demonstrating the robust *in vivo* anti-tumor immune responses upon 4-1BB triggering [6], [7]. This effect is largely interpreted by 4-1BB signaling on tumor-specific T cells that can enhance proliferation and CTL activity, and can prevent activation-induced cell death [8], [40]. Consistently, co-cultures of HDACi-treated Raji cells with T cell receptor-stimulated peripheral blood leukocytes enhanced T cell proliferation as compared to untreated Raji cells. This proliferative response was blocked in the presence of competing 4-1BB-Fc, but not by DR3-Fc recombinant protein, showing that 4-1BB/4-1BBL interaction was specifically required for this HDACi-enhanced T cell proliferative response. More recent studies have further revealed additional mechanisms that may account for 4-1BB anti-tumor effects. Firstly, 4-1BB signaling is able to prevent and rescue T cells from immune tolerance, promoting regression of poorly immunogenic tumors [54]. Secondly, 4-1BB expression on non-T cell populations, including natural killer cells and dendritic cells has been implicated [55], [56]. Finally, 4-1BB stimulation modifies the distribution pattern of tumor-specific T cells *in vivo*, in a manner that is largely dependent on IFNγ [57]. IFNγ, a pleiotropic cytokine secreted by activated T cells and NK cells, plays a central role in both the innate and adaptive immune response to a variety of pathogens and transformed cells [58]. CD4^+^ and CD8^+^ T cells have been shown to mediate tumor rejection in an IFNγ-dependent fashion through different mechanisms, including up-regulation of molecules critical for antigen processing and presentation [59], differentiation of fully competent effector cells [60], secretion of angiostatic chemokines by stromal cells present within a tumor [61], and regulation of T-cell migration to the tumor site [62]. In this study, co-cultures of MS-275-treated Raji cells with T cell receptor-stimulated peripheral blood leukocytes enhanced T lymphocyte IFNγ secretion, as compared to untreated Raji cells. Increased IFNγ secretion was blocked in the presence of competing 4-1BB/TNFRSF9-Fc recombinant protein, showing that 4-1BB/4-1BBL interactions were required. Importantly, induction of IFNγ was also observed in the absence of T cell activation, and was completely blocked in the presence of the 4-1BB-Fc recombinant protein, indicating that induction of IFNγ by MS-275-treated Raji cells occurred through 4-1BB triggering and could occur in the absence of T cell activation.

Despite similar levels of induction of 4-1BBL transcription and cell surface expression by TSA, SAHA and MS-275, only MS-275 potentiated IFNγ secretion in mixed leukocyte reactions and also provided more efficient T lymphocyte proliferative responses in these assays. These results suggest that compared to TSA and SAHA, MS-275 can provide additional specific signalling to improve further immune responses. Previous reports have shown that HDACi can induce the expression of CD86/B7.2 costimulatory molecule in acute myeloid leukemia (AML) cells and freshly isolated AML clinical samples [19]. Taken together with our present finding that HDACi can enhance expression of yet another costimulatory molecule, 4-1BBL, this data further supports HDACi, especially MS-275, as antitumor drugs to be used in immunotherapeutic clinical approaches. HDACi have been shown to induce growth arrest, differentiation, and/or apoptosis of cancer cells *in vitro* and in *vivo* tumor-bearing animals models [13], [63], [64]. In association with the proapoptotic effect of HDACi, the enhancement of costimulatory molecules on leukemia blasts could augment tumor immunogenicity, increasing specific CTL activity against tumor cells. Combinatorial treatment modulating both apoptotic and costimulatory molecules may be more effective for reinforcing host immunity and eradicating tumors. This point is supported by a recent study by Uno et al. who showed that induction of tumor cell apoptosis by an agonistic antibody to TRAILR2/DR5, combined with T cell activation by CD40 and 4-1BB agonistic monoclonal antibodies, potently and rapidly stimulated tumor-specific CD8^+^ T cells capable of eradicating pre-established tumors [65]. It is noteworthy that HDACi treatment did not induce 4-1BBL transcription in breast-derived tumor cells lines, nor did it induce significant 4-1BBL transcription in primary human peripheral blood lymphocytes, suggesting leukemia specific modulation of 4-1BBL by HDACi treatment (BV, data not shown).

In conclusion, we have demonstrated that HDACi can modulate the expression of 4-1BBL costimulatory molecule in B leukemia cell line model, which can enhance T cell responses towards the tumor cells. Further studies are now required to examine these effects using primary cells from patients with chronic lymphocytic leukemia (CLL) and to determine in animal models whether 4-1BBL/*TNFSF9* up-regulation contributes to HDACi-induced anti-tumor effects *in vivo*. The distinct capacity of MS-275 as compared to other HDACi to modulate these T cell responses will also require further investigation.

## References

[1] Hehlgans T, Pfeffer K (2005). The intriguing biology of the tumour necrosis factor/tumour necrosis factor receptor superfamily: players, rules and the games.. Immunology.

[2] Locksley RM, Killeen N, Lenardo MJ (2001). The TNF and TNF receptor superfamilies: integrating mammalian biology.. Cell.

[3] Moore RJ, Owens DM, Stamp G, Arnott C, Burke F (1999). Mice deficient in tumor necrosis factor-alpha are resistant to skin carcinogenesis.. Nat Med.

[4] Knight B, Yeoh GC, Husk KL, Ly T, Abraham LJ (2000). Impaired preneoplastic changes and liver tumor formation in tumor necrosis factor receptor type 1 knockout mice.. J Exp Med.

[5] Zerafa N, Westwood JA, Cretney E, Mitchell S, Waring P (2005). Cutting edge: TRAIL deficiency accelerates hematological malignancies.. J Immunol.

[6] Watts TH (2005). TNF/TNFR family members in costimulation of T cell responses.. Annu Rev Immunol.

[7] Melero I, Shuford WW, Newby SA, Aruffo A, Ledbetter JA (1997). Monoclonal antibodies against the 4-1BB T-cell activation molecule eradicate established tumors.. Nat Med.

[8] Shuford WW, Klussman K, Tritchler DD, Loo DT, Chalupny J (1997). 4-1BB costimulatory signals preferentially induce CD8+ T cell proliferation and lead to the amplification in vivo of cytotoxic T cell responses.. J Exp Med.

[9] Insinga A, Monestiroli S, Ronzoni S, Gelmetti V, Marchesi F (2005). Inhibitors of histone deacetylases induce tumor-selective apoptosis through activation of the death receptor pathway.. Nat Med.

[10] Nebbioso A, Clarke N, Voltz E, Germain E, Ambrosino C (2005). Tumor-selective action of HDAC inhibitors involves TRAIL induction in acute myeloid leukemia cells.. Nat Med.

[11] Drummond DC, Noble CO, Kirpotin DB, Guo Z, Scott GK (2005). Clinical development of histone deacetylase inhibitors as anticancer agents.. Annu Rev Pharmacol Toxicol.

[12] Minucci S, Pelicci PG (2006). Histone deacetylase inhibitors and the promise of epigenetic (and more) treatments for cancer.. Nat Rev Cancer.

[13] Saito A, Yamashita T, Mariko Y, Nosaka Y, Tsuchiya K (1999). A synthetic inhibitor of histone deacetylase, MS-27-275, with marked in vivo antitumor activity against human tumors.. Proc Natl Acad Sci U S A.

[14] Richon VM, Emiliani S, Verdin E, Webb Y, Breslow R (1998). A class of hybrid polar inducers of transformed cell differentiation inhibits histone deacetylases.. Proc Natl Acad Sci U S A.

[15] Richon VM, Webb Y, Merger R, Sheppard T, Jursic B (1996). Second generation hybrid polar compounds are potent inducers of transformed cell differentiation.. Proc Natl Acad Sci U S A.

[16] Yoshida M, Kijima M, Akita M, Beppu T (1990). Potent and specific inhibition of mammalian histone deacetylase both in vivo and in vitro by trichostatin A.. J Biol Chem.

[17] Kim MS, Kwon HJ, Lee YM, Baek JH, Jang JE (2001). Histone deacetylases induce angiogenesis by negative regulation of tumor suppressor genes.. Nat Med.

[18] Leoni F, Zaliani A, Bertolini G, Porro G, Pagani P (2002). The antitumor histone deacetylase inhibitor suberoylanilide hydroxamic acid exhibits antiinflammatory properties via suppression of cytokines.. Proc Natl Acad Sci U S A.

[19] Maeda T, Towatari M, Kosugi H, Saito H (2000). Up-regulation of costimulatory/adhesion molecules by histone deacetylase inhibitors in acute myeloid leukemia cells.. Blood.

[20] Magner WJ, Kazim AL, Stewart C, Romano MA, Catalano G (2000). Activation of MHC class I, II, and CD40 gene expression by histone deacetylase inhibitors.. J Immunol.

[21] Castellano R, Vire B, Pion M, Quivy V, Olive D (2006). Active transcription of the human FASL/CD95L/TNFSF6 promoter region in T lymphocytes involves chromatin remodeling: role of DNA methylation and protein acetylation suggest distinct mechanisms of transcriptional repression.. J Biol Chem.

[22] Osborn L, Kunkel S, Nabel GJ (1989). Tumor necrosis factor alpha and interleukin 1 stimulate the human immunodeficiency virus enhancer by activation of the nuclear factor kappa B.. Proc Natl Acad Sci U S A.

[23] Bradford MM (1976). A rapid and sensitive method for the quantitation of microgram quantities of protein utilizing the principle of protein-dye binding.. Anal Biochem.

[24] Van Lint C, Ghysdael J, Paras P, Burny A, Verdin E (1994). A transcriptional regulatory element is associated with a nuclease-hypersensitive site in the pol gene of human immunodeficiency virus type 1.. J Virol.

[25] Johnstone RW (2002). Histone-deacetylase inhibitors: novel drugs for the treatment of cancer.. Nat Rev Drug Discov.

[26] Levine M, Tjian R (2003). Transcription regulation and animal diversity.. Nature.

[27] Jin S, Scotto KW (1998). Transcriptional regulation of the MDR1 gene by histone acetyltransferase and deacetylase is mediated by NF-Y.. Mol Cell Biol.

[28] Kim S, Kang JK, Kim YK, Seo DW, Ahn SH (2006). Histone deacetylase inhibitor apicidin induces cyclin E expression through Sp1 sites.. Biochem Biophys Res Commun.

[29] Park SH, Lee SR, Kim BC, Cho EA, Patel SP (2002). Transcriptional regulation of the transforming growth factor b type II receptor gene by histone acetyltransferase and deacetylase is mediated by NF-Y in human breast cancer cells.. Journal of Biological Chemistry.

[30] Sowa Y, Orita T, Minamikawa S, Nakano K, Mizuno T (1997). Histone deacetylase inhibitor activates the WAF1/Cip1 gene promoter through the Sp1 sites.. Biochem Biophys Res Commun.

[31] Yokota T, Matsuzaki Y, Miyazawa K, Zindy F, Roussel MF (2004). Histone deacetylase inhibitors activate INK4d gene through Sp1 site in its promoter.. Oncogene.

[32] Zhang X, Wharton W, Yuan Z, Tsai SC, Olashaw N (2004). Activation of the growth-differentiation factor 11 gene by the histone deacetylase (HDAC) inhibitor trichostatin A and repression by HDAC3.. Mol Cell Biol.

[33] Kavurma MM, Bobryshev Y, Khachigian LM (2002). Ets-1 positively regulates Fas ligand transcription via cooperative interactions with Sp1.. J Biol Chem.

[34] Bhuiyan MPI, Kato T, Okauchi T, Nishino N, Maeda S (2006). Chlamydocin analogs bearing carbonyl group as possible ligand toward zinc atom in histone deacetylases.. Bioorganic & Medicinal Chemistry.

[35] Miller DM, Polansky DA, Thomas SD, Ray R, Campbell VW (1987). Mithramycin selectively inhibits transcription of G-C containing DNA.. Am J Med Sci.

[36] Liu F, Pore N, Kim M, Voong KR, Dowling M (2006). Regulation of histone deacetylase 4 expression by the SP family of transcription factors.. Mol Biol Cell.

[37] Suske G (2000). Transient transfection of Schneider cells in the study of transcription factors.. Methods Mol Biol.

[38] Gramaglia I, Cooper D, Miner KT, Kwon BS, Croft M (2000). Co-stimulation of antigen-specific CD4 T cells by 4-1BB ligand.. Eur J Immunol.

[39] Kwon BS, Weissman SM (1989). cDNA sequences of two inducible T-cell genes.. Proc Natl Acad Sci U S A.

[40] Takahashi C, Mittler RS, Vella AT (1999). Cutting edge: 4-1BB is a bona fide CD8 T cell survival signal.. J Immunol.

[41] Migone TS, Zhang J, Luo X, Zhuang L, Chen C (2002). TL1A is a TNF-like ligand for DR3 and TR6/DcR3 and functions as a T cell costimulator.. Immunity.

[42] Glick RD, Swendeman SL, Coffey DC, Rifkind RA, Marks PA (1999). Hybrid polar histone deacetylase inhibitor induces apoptosis and CD95/CD95 ligand expression in human neuroblastoma.. Cancer Res.

[43] Peart MJ, Smyth GK, van Laar RK, Bowtell DD, Richon VM (2005). Identification and functional significance of genes regulated by structurally different histone deacetylase inhibitors.. Proc Natl Acad Sci U S A.

[44] Ruefli AA, Ausserlechner MJ, Bernhard D, Sutton VR, Tainton KM (2001). The histone deacetylase inhibitor and chemotherapeutic agent suberoylanilide hydroxamic acid (SAHA) induces a cell-death pathway characterized by cleavage of Bid and production of reactive oxygen species.. Proc Natl Acad Sci U S A.

[45] Glaser KB, Staver MJ, Waring JF, Stender J, Ulrich RG (2003). Gene expression profiling of multiple histone deacetylase (HDAC) inhibitors: defining a common gene set produced by HDAC inhibition in T24 and MDA carcinoma cell lines.. Mol Cancer Ther.

[46] Mariadason JM, Corner GA, Augenlicht LH (2000). Genetic reprogramming in pathways of colonic cell maturation induced by short chain fatty acids: comparison with trichostatin A, sulindac, and curcumin and implications for chemoprevention of colon cancer.. Cancer Res.

[47] Mitsiades CS, Mitsiades NS, McMullan CJ, Poulaki V, Shringarpure R (2004). Transcriptional signature of histone deacetylase inhibition in multiple myeloma: biological and clinical implications.. Proc Natl Acad Sci U S A.

[48] Van Lint C, Emiliani S, Verdin E (1996). The expression of a small fraction of cellular genes is changed in response to histone hyperacetylation.. Gene Expr.

[49] Huang L, Sowa Y, Sakai T, Pardee AB (2000). Activation of the p21WAF1/CIP1 promoter independent of p53 by the histone deacetylase inhibitor suberoylanilide hydroxamic acid (SAHA) through the Sp1 sites.. Oncogene.

[50] Vigushin DM, Coombes RC (2004). Targeted histone deacetylase inhibition for cancer therapy.. Curr Cancer Drug Targets.

[51] Ammanamanchi S, Freeman JW, Brattain MG (2003). Acetylated sp3 is a transcriptional activator.. J Biol Chem.

[52] Camarero N, Nadal A, Barrero MJ, Haro D, Marrero PF (2003). Histone deacetylase inhibitors stimulate mitochondrial HMG-CoA synthase gene expression via a promoter proximal Sp1 site.. Nucleic Acids Res.

[53] Won J, Yim J, Kim TK (2002). Sp1 and Sp3 recruit histone deacetylase to repress transcription of human telomerase reverse transcriptase (hTERT) promoter in normal human somatic cells.. J Biol Chem.

[54] Wilcox RA, Flies DB, Zhu G, Johnson AJ, Tamada K (2002). Provision of antigen and CD137 signaling breaks immunological ignorance, promoting regression of poorly immunogenic tumors.. J Clin Invest.

[55] Wilcox RA, Chapoval AI, Gorski KS, Otsuji M, Shin T (2002). Cutting edge: Expression of functional CD137 receptor by dendritic cells.. J Immunol.

[56] Wilcox RA, Tamada K, Strome SE, Chen L (2002). Signaling through NK cell-associated CD137 promotes both helper function for CD8+ cytolytic T cells and responsiveness to IL-2 but not cytolytic activity.. J Immunol.

[57] Wilcox RA, Flies DB, Wang H, Tamada K, Johnson AJ (2002). Impaired infiltration of tumor-specific cytolytic T cells in the absence of interferon-gamma despite their normal maturation in lymphoid organs during CD137 monoclonal antibody therapy.. Cancer Res.

[58] Kaplan DH, Shankaran V, Dighe AS, Stockert E, Aguet M (1998). Demonstration of an interferon gamma-dependent tumor surveillance system in immunocompetent mice.. Proc Natl Acad Sci U S A.

[59] Boehm U, Klamp T, Groot M, Howard JC (1997). Cellular responses to interferon-gamma.. Annu Rev Immunol.

[60] Fallarino F, Gajewski TF (1999). Cutting edge: differentiation of antitumor CTL in vivo requires host expression of Stat1.. J Immunol.

[61] Qin Z, Blankenstein T (2000). CD4+ T cell—mediated tumor rejection involves inhibition of angiogenesis that is dependent on IFN gamma receptor expression by nonhematopoietic cells.. Immunity.

[62] Nakajima C, Uekusa Y, Iwasaki M, Yamaguchi N, Mukai T (2001). A role of interferon-gamma (IFN-gamma) in tumor immunity: T cells with the capacity to reject tumor cells are generated but fail to migrate to tumor sites in IFN-gamma-deficient mice.. Cancer Res.

[63] Butler LM, Agus DB, Scher HI, Higgins B, Rose A (2000). Suberoylanilide hydroxamic acid, an inhibitor of histone deacetylase, suppresses the growth of prostate cancer cells in vitro and in vivo.. Cancer Res.

[64] Jaboin J, Wild J, Hamidi H, Khanna C, Kim CJ (2002). MS-27-275, an inhibitor of histone deacetylase, has marked in vitro and in vivo antitumor activity against pediatric solid tumors.. Cancer Res.

[65] Uno T, Takeda K, Kojima Y, Yoshizawa H, Akiba H (2006). Eradication of established tumors in mice by a combination antibody-based therapy.. Nat Med.

